# The clinical value of cytokines in chronic fatigue syndrome

**DOI:** 10.1186/s12967-019-1948-6

**Published:** 2019-06-28

**Authors:** Tiansong Yang, Yan Yang, Delong Wang, Chaoran Li, Yuanyuan Qu, Jing Guo, Tianyu Shi, Wang Bo, Zhongren Sun, Tetsuya Asakawa

**Affiliations:** 1 First Affiliated Hospital, Heilongjiang University of Chinese Medicine, 26 Heping Road, Harbin, China; 2 Heilongjiang University of Chinese Medicine, 24 Heping Road, Harbin, China; 3 Department of Neurosurgery, Hamamatsu University School of Medicine, Handayama, 1-20-1, Higashi-ku, Hamamatsu, Shizuoka 431-3192 Japan; 4 Research Base of Traditional Chinese Medicine Syndrome, Fujian University of Traditional Chinese Medicine, Fuzhou, 350122 China

**Keywords:** Chronic fatigue syndrome, Inflammatory reaction, Cytokine, Individual diagnosis, Immune modulation

## Abstract

Chronic fatigue syndrome (CFS) is a heterogeneous disorder with uncertain pathogenesis. Without effective therapy, CFS is characterized by disabling fatigue, depression, memory loss, and somatic discomfort. This comprehensive and impartial review aimed to assess the available evidence and examined the potential clinical value of using cytokines for the monitoring of CFS and as targets for the treatment of CFS. Inflammatory reactions and immune modulation are considered to contribute to the pathophysiology of CFS, and it is well documented that cytokines present in both blood and cerebrospinal fluid (CSF) are closely associated with the progression and severity of CFS. However, pathophysiological and methodological limitations prevent using circulating cytokines as independent diagnostic indices. Moreover, there is no evidence to support the use of CSF cytokines as independent diagnostic indices. Nevertheless, a comprehensive evaluation of changes in circulating and CSF cytokines may improve clinical understanding of the pathophysiology of patients with CFS, aiding in the establishment of an appropriate diagnosis. Importantly, the available evidence does not support the value of cytokines as therapeutic targets. We believe that an improved understanding of cytokine-related mechanisms will be helpful to explore new cytokine-related therapeutic targets.

## Background

Chronic fatigue syndrome (CFS), also known as myalgic encephalomyelitis (ME), was first reported in 1988 [1]. It is defined as chronic and debilitating fatigue, which cannot be relieved by sufficient rest. Many other indications, such as flu-like symptoms, depression, sleep disturbance, poor concentration, memory loss, post-exertional malaise, and gastrointestinal dysfunction, are also associated with CFS. CFS is a highly complex disorder; however, it lacks satisfactory diagnosis and treatment. Its prevalence is approximately 0.2–0.3% [2], and it mostly affects women [3]. CFS is an increasing public health concern, making the development of sensitive and reliable biomarkers by clinicians an urgent requirement.

Mechanisms underlying CFS are complicated and not fully understood; it has been hypothesized that immune-inflammatory, neuroendocrine, and bioenergy metabolisms play predominant roles. Tomas et al. reported that patients with CFS, particularly women, usually suffer from hypothalamic–pituitary–adrenal (HPA) axis dysfunction [4].

Morris et al. have reported that HPA axis hypofunction in ME/CFS may be triggered by multiple immune-inflammatory mechanisms and may play a role in the pathogenesis of CFS by activating immune-inflammatory oxidative and nitrosative stress (O&NS) pathways and causing hypocortisolism [5]. Another study has suggested that mitochondrial dysfunction is also associated with immune inflammation, which can be induced by increased pro-inflammatory cytokines along with O&NS, and can lead to the onset of CFS [6]. A study reported that in patients with CFS, cellular energy metabolism does not meet the requirements for achieving mitochondrial stress responses under conditions of both basic and high metabolic demand [7]. The findings of these prior investigations support a prominent role of immune-inflammatory pathways in the pathogenesis of CFS.

Infection caused by viruses and bacteria is an important factor that induces fatigue via triggering immuno-inflammatory pathways. Previous studies have reported CFS as a common symptom after viral infection; those studies investigated numerous viruses, including Epstein–Barr virus [8, 9], herpesvirus-6 [10], parvovirus B19 [11, 12], and xenotropic murine leukemia virus-related virus [13]. Morris et al. have reported that bacterial infections caused by the translocation of intestinal microbiota play a role in the onset of CFS [14]. Subsequent research reported that despite the high heterogeneity among different CFS studies, enrolled patients shared the same inflammatory signs and symptoms, namely “sickness behavior (SB),” which includes fatigue, fever, myalgia and some other CFS symptoms and is mediated by proinflammatory cytokines [15]. SB is considered as an evolutionarily adaptive behavioral response to infection; once an individual is exposed to an immune-infective environment, inflammatory pathways are activated and a cytokine-mediated mechanism is triggered. This procedure may minimize energy consumption, which aids in an individual’s ability to cope with environmental risks. In situations wherein the infective environment is permanent, SB persists and induces chronic fatigue [16].

Underlying immune impairment is another potential mechanism associated with the immune-inflammatory mechanisms involved in CFS. Decreased cytotoxicity of natural killer (NK) and T cells, increased activity of B cells, and changes in cytokine profiles are always observed in CFS-related studies. A recent study has suggested that CFS can be defined as an autoimmune disease and that B cell depletion therapy with an anti-CD20 antibody (rituximab) is beneficial for the treatment of CFS [2, 17].

All the above-mentioned findings indicate that CFS is an immune-infection-related disorder. It is acknowledged that cytokines are important modulators of immune function and inflammatory response. Cytokines are very sensitive to changes in the immune system. It is well documented that the cytokine profile is altered in patients with CFS specifically, the enhancement of pro-inflammatory cytokines is commonly found in the affected patients. Furthermore, a number of studies have reported that a T helper 2 (Th2) cell-mediated mechanism is involved in the disease [18–20]. Cytokines are reportedly closely associated with CFS; however, the value of cytokines as biomarkers for the diagnosis of CFS remains controversial. Blundell et al. conducted a systematic review investigating the correlation between circulating cytokines and CFS and reported that circulating transforming growth factor-beta (TGF-β) levels were increased in patients with CFS and that no other cytokines exhibited a significant difference between patients with CFS and controls. However, the poor quality of the performed studies precluded robust conclusions [21]. Montoya et al. subsequently performed a systematic review with 192 patients with CFS and observed an increase in TGF-β levels and a decrease in resistin levels in the examined patients. Of the 17 cytokines correlated with CFS severity, only CXCL9 exhibited a negative correlation with fatigue duration [22]. A recent study by VanElzakker totally rejected the value of circulating cytokines as biomarkers for CFS owing to methodological weaknesses across the neuroimaging literature; the author suggested that mechanisms of both cytokine assessment and methodological confounders must be considered before comparing cytokine studies. He also reported a limited value of using such circulating cytokines in CFS studies with a within-subject or mixed-model challenge-type experimental design [23]. We agree with previous studies that have reported that cytokines are sensitive and easily affected by several factors, which are responsible for the high heterogeneity observed among studies. Randomized controlled trials that have investigated circulating cytokines and CFS are lacking; hence, any conclusions from the limited available evidence should be derived cautiously. In the present study, based on a comprehensive and balanced perspective, we have attempted to present a narrative review of the studies available in the literature to discuss the relationship and interactions between cytokines and CFS. The role of cytokines as potential diagnostic markers for CFS subgroups has also been addressed in an attempt to contribute to a better understanding of the role of these cell-signaling molecules in CFS (Table 1).

**Table 1:** Characteristics of the involved studies

References	Participants (n)	Age (years)	Course (years)	Samples	Findings
Moneghetti [45]	CFS/ME P (24)	46.3 ± 10.9	NA	B	The most discriminatory cytokines between ME/CFS cases and controls post exercise were serum CD40L, PAI-1, IL1-β, IFN-α and CXCL1
	HC (24)	41.6 ± 10.7			
Milrad [26]	CFS P (242)	49.36 ± 10.9	NA	B	Higher evening cortisol predicted greater depressive symptoms and circulating pro-inflammatory cytokines IL-2, IL-6, and TNF-α in CFS patients
	HC (392)	50.1 ± 12.5			
Clark [54]	CFS P (24)	40. 3 ± 12.2	NA	B	Serum TGF-β increased in patients compared to controls at rest; no difference of cytokines IL-2, IL-4, IL-5, IL-10, IL-12, p70, and IFN-γ between cases and controls
	HC (21)	39. 3 ± 14.1			
Milrad [3]	CFS/ME P (60)	50.52 ± 10.88	NA	B	Poor sleep quality is associated with enhancements of IL-1β, IL-6 and h fatigue severity
Montoya [22]	CFS/ME P (192)	49.9 ± 12.7	NA	B	Cytokines CCL11, CXCL1, CXCL10, IFN-γ, IL-4, IL-5, IL-7, IL-12p70, IL-13, IL-17F, leptin, G-CSF, GM-CSF, LIF, NGF, SCF, and TGF-α had a statistically significant upward linear trend correlated with ME/CFS severity
Russell [37]	Age ≤ 18 years ME/CFS P (18)	15.78 + 1.69	2.0 + 0.0	B	Plasm IL-1α increased in recently ill adolescent ME/CFS subjects, and was progressively less important with duration; IL-8 increase screened positive for ME/CFS in the recently afflicted, the opposite was true for subjects ill for more than 2 years; IL-6 decrease suggested early ME/CFS, the reverse was true in subjects over 18 years of age ill for more than 2 years
	18 < Age ≤ 50 years ME/CFS P (22)	40.82 + 6.17	7.1 + 6.0		
	Age > 50 years ME/CFS P (28)	60.21 + 6.66	10.6 + 7.7		
	HC (73)	14-60			
Hornig [80]	CFS/ME P (32)	44.2 ± 7.1	7.6 ± 7.3	C	Cytokines IL-1ra, IL-1β, IL-5, IL-6, IL-8, IL-10, IL-12p40, IL-17F, TNF-β, SCF, CSF1, CSF2, CSF3, PDGFBB, FGFb, VEGFA, LIF, resistin, serpin E1, sICAM1 and VCAM1 decreased; CCL11 and CXCL10 increased in CFS patients in comparison to no disease control
	MS P (40)	49.9 ± 11.4			
	HC (19)	50.5 ± 8.5			
Hornig [33]	Short-duration ME/CFS P (52)	40.05 ± 13.6	1.7 ± 0.8	B	Plasm IL-1α, CXCL8, IL-12p40, IL-17A, TNFα, sFasL, TRAIL, CCL2, SCF, resistin, IL-1RA and IL-13 increased; CD40L and PDGFBB decreased in short duration CFS patients versus long duration CFS patients and control
	Long-duration ME/CFS P (246)	50.02 ± 11.4	15.6 ± 8.2		
	HC (348)	48.5 ± 12.0	Blank control		
Peterson [79]	CFS P (18)	NA	NA	C	IL-10 decreased in the CFS/ME patients in comparison to the controls
	HC (15)	NA			
Hardcastle [34]	Severely CFS/ME P (19)	40.21 + 1.57	13.071 + 6.639	B	Serum IL-1β and RANTES decreased, IFN-γ increased in severe compared with moderate CFS/ME patients; IL-6 decreased in moderate CFS/ME patients compared with healthy controls and severe CFS/ME patients; IL-7and IL-8 increased in the severe CFS/ME group compared with healthy controls and moderate CFS/ME patients
	Moderately CFS/ME P (22)	42.09 + 2.72	9.00 + 8.870		
	HC (22)	40.14 + 2.38			
Maes [84]	CF P (37)	41.6 ± 11.5	NA	B	Plasma IL-1 and TNF-α increased, serum neopterin increased in CFS and ME than in CF patients
	CFS P (58)	39.1 ± 13.2			
	MEP (49)	43.7 ± 13.1			
Brenu [50]	CFS/ME P (65)	47.2 ± 11.5	16.4 ± 12.5	B	Cytokines IL-10, IFN-γ and TNF-α increased at baseline; IL-10 and IL-17A decreased at 6 months; IL-2 increased at 12 months in the CFS/ME group in comparison to the non-fatigued controls
	HC (21).	45.2 ± 9.3			
Brenu [18]	CFS/ME P (95)	46.47 ± 11.7	NA	B	CFS/ME patients displayed IL-10, IFN-γ and TNF-α enhancement in PBMCs
	HC (50)	41.9 ± 9.6			
Broderick [19]	CFS P (40)	50	NA	B	Circulating level of IL-1a, 1b, IL- 4, IL- 5, IL- 6, IL- 12 and LTα increased; IL-8, IL-13, and IL-15 decreased; no difference of IL-2, 10, 17, IL-23, IFN-γ, and TNF-α was found in CFS patients compared with controls
	HC (59)	53			
Fletcher [43]	CFS P (40)	50	NA	B	Plasma LTα, IL-1α, IL-1β, IL-4, IL-5, IL-6 and IL-12 increased, IL-8, IL-13 and IL-15 decreased, no difference of TNF-α, IFN-γ, IL-2, IL-10, IL-23 and IL-17 was found when comparing CFS with controls
	HC (59)	53			
Nater [36]	CFS P (28)	49.6	NA	B	Changes of diurnal salivary cortisol rhythm were identical with IL-6 enhancement in CFS cases compared with the other groups
	Persons with ISF (35)	49.2			
ter Wolbeek [48]	CFS P (11)	15.91 ± 1.34	0.62 ± 0.32	B	Anti-inflammatory cytokines IL-10 increased (IFN-γ/IL-10 decreased), pro-inflammatory cytokines IL-6, and TNF-a decreased in CFS patients when compared with the severely fatigued or non-fatigued participants
	Severely fatigued controls (67)	15.18 ± 1.37			
	Non-fatigued controls (61)	14.74 ± 1.60			
Natelson [78]	CFS P (44)	41.4 ± 8.0	NA	C	GM-CSF decreased in patients in comparison to the controls; IL-8 increased in patients with sudden, influenza-like onset than in patients with gradual onset or in controls; IL-10 increased in the patients with abnormal spinal fluids than in those with normal fluid or controls
	HC (13)	33.0 ± 10.5			
Zhang [52]	Veterans CFS (43)	47.2 ± 11.5	NA	B	IL-2, IL-10, IFN-γ, and TNF-α increased in veterans with CFS compared with controls; there were no changes between civilians with CFS and controls
	Veterans control (34)	NA			
	Civilian CFS (68)	NA	NA		
	Civilian control (53)	NA			
Borish [49]	CFS patients (18)	43.3	NA	B	TNF-α increase in PBMCs is observed in CFS group and allergic group but not in depression group compared with control group. IL-10 decrease in PBMCs in CFS group, allergic group and depression group compared with control group
	HC (11)	42.9			
	Allergic control subjects (14)	43.3			
	Depression control subjects (12)	45.8			
Vojdani [30]	CFS P (29)	17–71	1–5	B	IFN-α increased in patients with CSF in plasma and cell lysate
	HC (15)	17–60			

*B* blood, *C* cerebrospinal fluid, *PBMCs* peripheral blood mononuclear cell, *LTα* lymphotoxin-alpha, *GM-CSF* granulocyte-macrophage colony-stimulating factor, *PAI-1* plasminogen activator inhibitor, *sFasL* soluble Fas ligand, *IL-1RA* IL-1 receptor antagonist, *CD40L* CD40 ligand, *MS* multiple sclerosis, *ISF* insufficient symptoms or fatigue (for CFS diagnosis)

## Search strategy

**Fig. 1:**
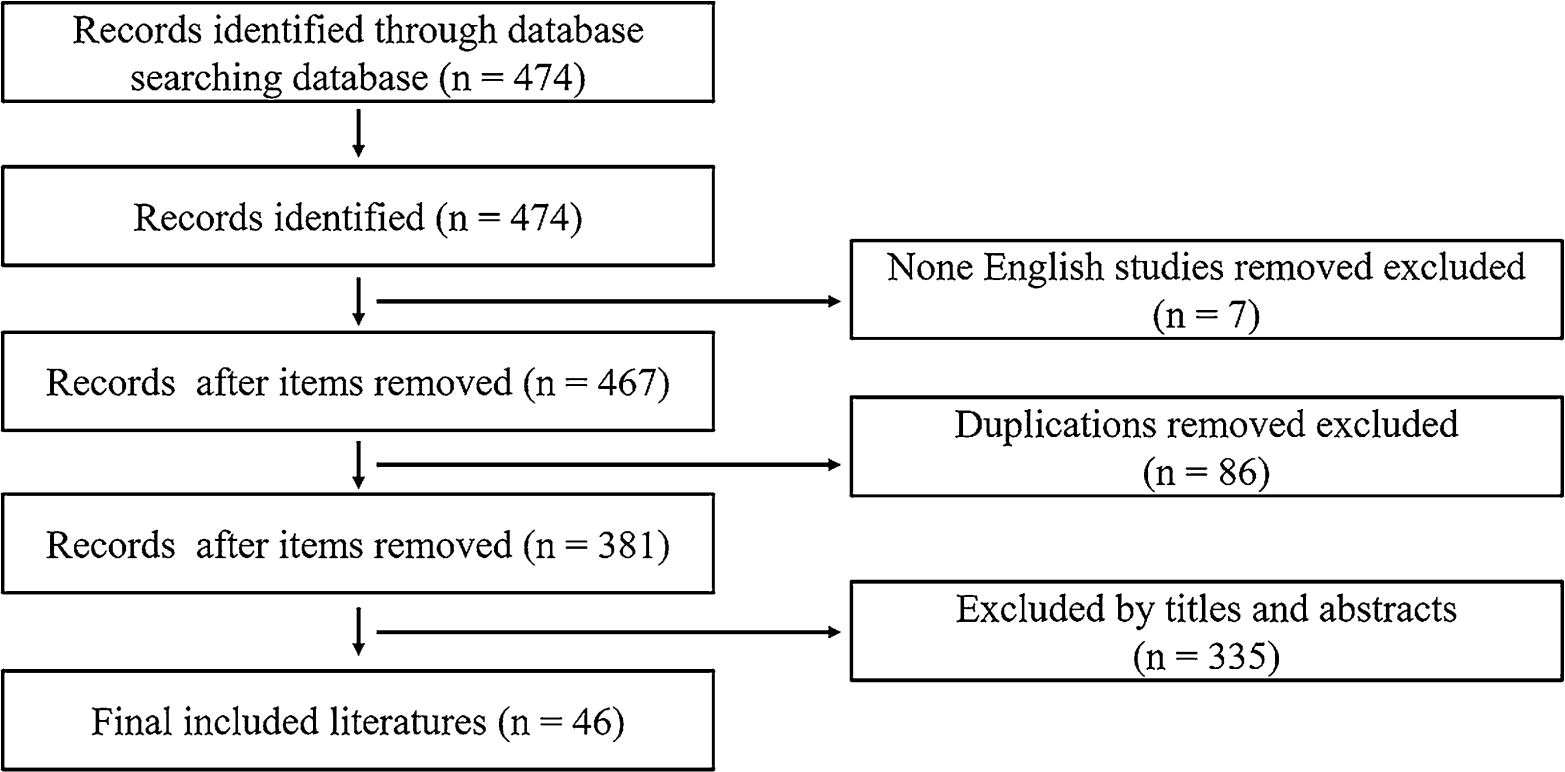
Flow chart of search strategy and selection of the literatures

We conducted an English-language search of databases, including PubMed, EMBASE, Web of Science, and Google Scholar, by using the terms “Chronic fatigue syndrome” OR “myalgic encephalomyelitis” AND “cytokine” OR “Tumor necrosis factor-α” OR “Interferons” OR “Interleukins” OR “TGF-β”. Literature from 1988–Feb 2019 was included. Basically, peer-reviewed original studies and review papers in English were considered. Literatures obtained by searching the above-mentioned databases were seriously read to identify additional reports. All the studies involving CFS and cytokine were included. The final references were established using citations in the context of the present review. The search strategy was shown as Fig. 1.

## Blood cytokines in CFS

Since CFS was defined as a disease entity, investigation of serum cytokines in these patients has been commonly performed owing to the quick availability and low invasiveness of blood samples. Despite these studies exhibiting high heterogeneity, it is worth summarizing the value of these circulating cytokines as a biomarker for diagnosing and evaluating CFS severity.

### Tumor necrosis factor-α (TNF-α)

TNF-α is a proinflammatory molecule with antitumor and antiviral effects and is thought to play a role in the pathogenesis of acquired immune deficiency syndrome and multiple sclerosis. It is the most commonly studied cytokine in the majority of CFS studies. However, the relationship between TNF-α and CFS remains controversial. Chao et al. established a cellular model of CFS by simulating peripheral blood mononuclear cells (PBMCs) with lipopolysaccharides. They reported increased levels of TNF-α in the model [24]. Another study found the similar result in non-adherent lymphocytes [25]. As for in vivo evidence, Milrad et al. reported that poor sleep quality was associated with increased levels of TNF-α and symptom severity in patients with CFS [3]. The authors reported that TNF-α levels are positively correlated with depressive symptoms in patients with CFS [26]; however, conflicting results were reported by few studies. Lidbury et al. found no significant differences in the circulating TNF-α levels between patients with CFS and controls [27]. Groven et al. reported that patients with CFS have higher TNF-α levels than healthy controls; however, the difference was not statistically significant (p = 0.056). TNF-α levels exhibited a weaker association with depression in patients with CFS than in healthy controls [28]. However, these studies did not corroborate with the results that TNF-α levels are positively correlated with CFS symptoms, probably owing to differences in the experimental conditions and study design, such as in vivo data used, small sample size, and different diagnostic criteria. Thus, we support the hypothesis that serum TNF-α is a relevant marker for the diagnosis of CFS.

### Interferons (IFNs)

Interferons (IFNs) can be categorized into two subtypes, namely, type I interferons, which include IFN-α and IFN-β, and type II interferons, which include IFN-γ. Both subtypes influence the development of CFS. Interestingly, in some patients undergoing treatment with IFN-α/β, a primary complaint is “severe fatigue” [29]; this is regarded as a major side effect of IFN-α/β. This may be an indirect evidence of a relationship between IFNs and CFS. In 1997, Vojdani reported upregulated circulating IFN-α levels accompanied by activated protein kinase R and induction of apoptosis in patients with CFS [30]. A subsequent study reported that the dysregulation of type I interferon pathway results in sustained upregulation of 2′-5′-oligoadenylate synthetase, which possibly contributes to the development of fatigue symptoms associated with CFS [29]. IFN-γ triggers several antiviral mechanisms related to chronic infection [31]. A comparative study demonstrated enhanced and persisting circulating TNF-α and IFN-γ levels in patients with CFS, similar to that observed in patients with acute B19 infection [32]. Another study reported significantly higher IFN-γ levels in PBMCs isolated from patients with CFS than from healthy individuals [18]. However, the immunopathology of CFS is not static, and the cytokine profile changes along the course of the disease. Differences in IFN-γ have been reported in patients with both short-and long-term disease. Markedly elevated IFN-γ levels have been associated with the early phase of CFS [33]. Moreover, IFN-γ is closely associated with CFS severity. Hardcastle reported that IFN-γ levels are higher in patients with severe CFS than in those with moderate disease [34]. Further, Montoya et al. reported that IFN-γ levels exhibit a significant linear upward correlation with CFS severity [22]. The above-mentioned evidence indicates that IFN-γ, rather than IFN-α/β, is a good biomarker in the early stage of CFS and that it can be used for clinical decisions regarding CFS severity.

### Interleukins (ILs)

#### IL-6

Because CFS is associated with HPA axis dysfunction, IL-6, an important inflammatory cytokine and HPA axis modulator [35], is considered to be associated with CFS. Plasma IL-6 levels are reportedly higher in patients with CFS than in healthy controls [19, 24, 36]. Plasma IL-6 levels exhibit a dose–effect relationship with CFS severity. Hardcastle et al. reported increased plasma IL-6 levels in patients with severe CFS and relatively lower levels of it in those with moderate CFS [34]. Another study reported dynamic changes in IL-6 levels along CFS progression, including lower levels in early CFS stages and increased levels with disease progression [37]. Moreover, IL-6 reportedly plays an important role in the main symptoms of CFS, such as hyperalgesia, fatigue, sleep impairment, and depression. Wallace et al. reported that increased IL-6 levels contribute to the pathogenesis of fibromyalgia, the main symptoms of which are chronic diffuse muscle pain, fatigue, and skin sensitivity [38]. Yoshimura et al. demonstrated that plasma IL-6 levels reflect major depressive disorder severity [39]. In addition, a recent study found a positive correlation between plasma IL-6 levels and depression severity in patients with CFS [26]. IL-6 reportedly induces excessive daytime sleepiness or disturbed non-refreshing sleep in patients with CFS. Increased IL-6 levels are associated with worse sleep quality [3, 40]. One study concerning the impact of emotional distress on CFS has suggested that IL-6 may comprise a plausible subgroup cytokine “biomarker” for CFS, as the enhancement of IL-6 levels in CFS patients showed positive effects with respect to emotional distress and symptom exacerbation [41]. Overall, these findings suggest that plasma IL-6 levels are an important biomarker for the diagnosis and management of CFS severity.

#### IL-1

IL-1 acts not only as a proinflammatory cytokine but also as a major mediator in central fatigue pathways [42]. IL-1 comprises the two subgroups IL-1α and IL-1β. IL-1β is frequently involved in CFS-related studies. Increased IL-1α and IL-1β levels have been reported in female patients with CFS than in healthy controls [43]. Hardcastle et al. found lower IL-1β levels in patients with severe CFS than in those with moderate CFS [34]. Several studies have reported that the enhancement of IL-1 in CFS is usually accompanied by changes in other cytokines [3, 24]. Maes et al. observed higher IL-1, TNF-α, and neopterin levels in patients with CFS than in those with chronic fatigue; hence, IL-1, TNF-α, and neopterin can be used as biomarkers to distinguish between the two conditions [44]. A recent study found that CD40L, PAI-1, IL1-β, IFN-α, and CXCL1 are the most discriminatory cytokines for post-exercise fatigue in patients with CFS [45].

#### IL-10

IL-10 is an anti-inflammatory cytokine secreted by Th2 cells. Increased IL-10 levels suggest decreased cytotoxic activity, as observed during the stages of persistent chronic infection [46]. However, the biological significance of serum IL-10 is controversial. Most authors believe that IL-10 levels are higher in patients with CFS than in healthy controls [18, 47]. A longitudinal study reported increased IL-10 levels accompanied by decreased IFN-γ/IL-10 ratio in patients with CFS during a 1-year observation period [48]. Nijs reported that exercise upregulates IL-10 expression in CFS patients at the gene level, but not protein level [47]. However, few studies have contradicted these findings [25, 49]. In a longitudinal research, IL-10 was assessed in PBMCs of patients with CFS and in healthy controls; IL-10 levels exhibited a significant increase at baseline, followed by a significant drop at 6 months and finally no significant variation at 12 months [50]. Thus, the role of IL-10 in CFS requires further investigation.

#### IL-2

IL-2 is a proinflammatory T cell growth factor secreted by Th1 cells. IL-2 contributes to the promotion of T cell proliferation as well as the restoration and proliferation of NK cells [19, 51]. Increased IL-2 levels in blood were confirmed by both clinical and bench studies [52, 53]. A recent study reported an enhancement of circulating pro-inflammatory cytokines (including that of IL-2, IL-6, and TNF-α), which was associated with depression in patients with CFS [26]. However, some studies have contradicted these results, with no significant differences in IL-2 levels between patients with CFS and healthy controls [43, 54]. Consequently, the value of IL-2 as a biomarker for CFS remains to be validated.

#### Other ILs

The roles of other ILs in this context remain poorly understood and require more investigation. Current evidence indicates that ILs play modulatory roles by interacting with other cytokines. IL-4 is a key factor that induces Th2 cell differentiation. In one report, the levels of IL-4 and those of IL-5, another Th2 marker, were found to be enhanced in CFS [22]. A Th17-related mechanism has also been reported in CFS; the mechanism considers Th17 a key player in inflammatory and autoimmune regulation. Th17 is closely associated with the pathophysiology of CFS [55]. Broderick et al. reported the co-expression patterns of cytokines IL-2, IL-6, IL-8, IL-23, and IFN-γ may be useful as potential biomarkers to distinguish adolescents in whom CFS does or does not develop after infection [56]. IL-12 is known to stimulate IFN-γ and TNF-α production by NK and T cells. This effect can be primarily enhanced by IL-2 and less by IL-4. Circulating IL-12 levels are reportedly higher in patients with CFS than in healthy controls [19]. IL-7 is associated with the activation of chronic immune response, which closely relates to CFS. Serum IL-7 and IL-8 reportedly display a statistically significant upward linear correlation trend with CFS severity [34]. IL-8 is also reportedly associated with the disease duration, with higher IL-8 levels observed in recently ill patients with CFS [37].

Although the functions and action mechanisms of each IL are different and multifactorial, enhancement of ILs is seemingly associated with CFS onset. Higher levels of ILs suggest a higher severity of CFS and its symptoms and a more advanced stage of disease progression. IL-6 and IL-1 may be useful biomarkers for CFS, whereas remaining ILs require further investigation.

### TGF-β

TGF-β is a multifunctional cytokine involved in several biological activities, such as cell-cycle control, hematopoiesis, angiogenesis, chemotaxis, and immune responses. Many TGF-β isoforms have proven to be associated with immune and neuroendocrine regulation in patients with CFS. TGF-β1 plays an inhibitory role in macrophages and NK cells, in the proliferation of T or B cells, and in the maturation of cytotoxic T lymphocytes [57]. TGF-β3 has been suggested to partly mediate the association between plasma cortisol and downregulation of expression of some B cell genes [58]. A systematic review concluded that TGF-β levels were enhanced in patients with CFS in most studies [21]. Montoya et al. reported higher TGF-β levels in patients with CFS than in healthy controls, independent of CFS severity [22]. In addition, activin B, as a member of the TGF-β family, has been suggested as a novel biomarker due to its significantly increased levels in CFS patients [27]. However, recently, few studies have reported unchanged TGF-β levels in patients with CFS [58, 59]. The relationship between TGF-β and CFS and the value of alterations in the TGF-β levels require further investigation in the future.

In this section, we discussed the potential value of using circulating cytokines as diagnostic biomarkers for CFS. The main strengths of such an approach lie in the easy sample collection, sensitivity, and reduced invasiveness. However, several limitations must be considered with respect to this approach: (1) Because of the blood–brain barrier (BBB), changes in peripheral cytokines sometimes may not represent the changes in the central nervous system (CNS). In patients with CFS, activated cytokines might be confined to the brain [60]. (2) Measurement of circulating cytokines is technically complicated. Although cytokine indices are sensitive, they are easily affected by many factors; therefore, proper sample handling is critical. (3) Systematic errors may arise when using circulating cytokines: for example, Roerink et al. have reported that most cytokines remain in the intercellular environment, where actual cytokine levels (particularly those of IL-1) are generally below the threshold of detection [60], but in a later study of the same authors found that the normalized protein expression value of IL-12p40 and CSF-1 was significantly higher in patients with CFS [61]. Therefore, we believe that the values of circulating cytokines must be defined as “indirect” and “auxiliary.” Thus far, they cannot be employed as “independent diagnostic biomarkers” for CFS and should be used in conjunction with other indices for the diagnosis of CFS. We conclude that further investigation of cytokines in the CNS of CFS patients is indispensable.

## The value of cytokines in the CSF

### Inflammatory changes in CNS during CFS

CFS is a disorder that involves the CNS. Neuropsychological symptoms, such as depression and anxiety, are commonly observed in patients with CFS. A previous meta-analysis has reported that the primary cognitive problems in patients with CFS are deficits of attention, memory, and reaction time [62]. This result is in agreement with the findings of a study that involved 307 cases [63]. Clinically, cognitive behavior therapy (CBT) has been used to treat CFS patients [64]. Brain imaging research has also demonstrated CNS abnormalities in patients with CFS. Reduced gray matter volumes have been well documented in patients with CFS [65, 66]. A longitudinal MRI study has found a significant reduction in white matter volumes in the left inferior fronto-occipital fasciculus in patients with CFS [67]. These findings confirm the role of central neural mechanisms in the onset of CFS.

These alterations in brain structure and function can be explained by a variety of complex mechanisms related to CNS inflammation [68, 69]. It is well documented that inflammation in CNS plays a key role in the pathophysiology of CFS [56]. Nakatomi et al. employed positron emission tomography to investigate the inflammatory changes occurring in the brain during CFS. A wide range of inflammatory changes were observed in the cingulate cortex, thalamus, midbrain, hippocampus, amygdala, and pons in patients with CFS. Additionally, this study pointed out the range of cerebral inflammation was highly associated with the severity of neuropsychological symptoms [70]. Chan et al. also reported that abnormal hippocampal neurogenesis is related to depression in CFS [71].

All the retrieved evidence suggests that inflammation in CNS is responsible for the pathophysiological changes in CFS, with regional symptoms being coherent with involved brain areas.

**Fig. 2:**
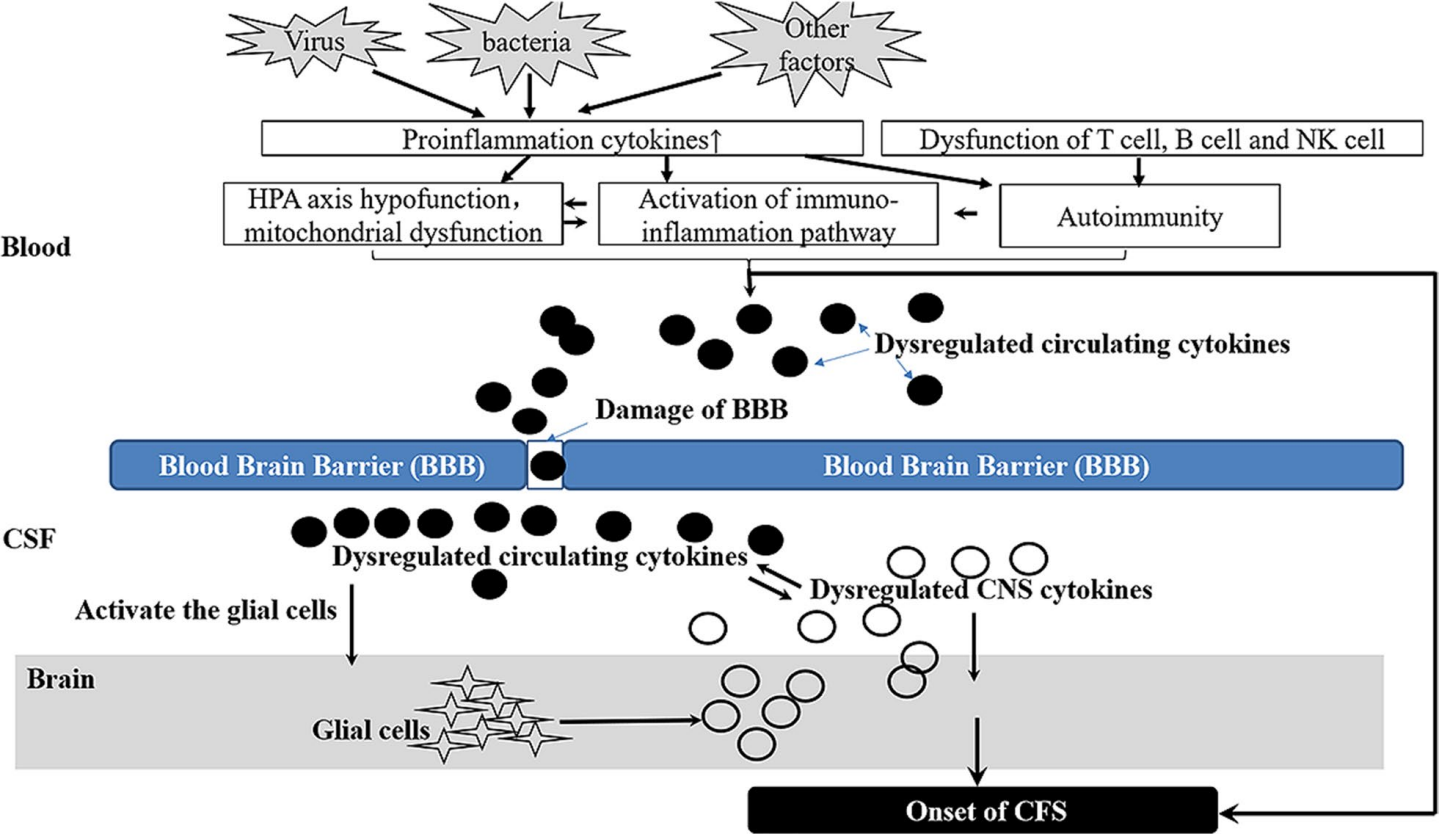
Potential cytokine-related mechanisms of CFS

In normal states, lymphocytes and their products are rarely found in the parenchyma of CNS because of the BBB action. However, in inflammatory states, BBB is damaged due to the effects of pro-inflammatory cytokines [72]. IL-1β exclusively attacks the paracellular barrier through the breakdown and translocation of tight junction proteins, and TNF-α targets the transcellular processes mediated by caveolae. A number of reactive oxygen and nitrogen species, accompanied by inflammatory reaction, also trigger BBB damage [73]. In the context of a damaged BBB, circulating cytokines can enter the CNS and activate glial cells (astrocytes, microglia, and oligodendrocytes) in the brain, promoting the secretion of cytokines, such as TNF-α, IL-I, and IL-6, in CNS (Fig. 2) [74]. One psycho-neuro-immunological hypothesis is that once the vagus nerve is infected, it becomes very sensitive to proinflammatory cytokines; it then communicates this cytokine exposure to the brain, thus, initiating the inflammatory components of CFS [75, 76].

### Cytokines in CSF

It is commonly known that CSF can reflect biochemical changes in the extracellular brain space. Because of the limitations of peripheral cytokines. It is reasonable to consider whether exploring cytokine changes in CSF can be used as a potential diagnostic biomarker, as well as an important tool to understand the underlying mechanisms of inflammation in CNS related to CFS. Many studies reported changes in pro-inflammatory biomarkers in CSF of patients with CFS. Early in 1991, Lloyd et al. have compared cytokine levels between patients with CFS and normal controls, revealing that IFN-α levels in CSF were enhanced in CFS patients [77]. Natelson et al. investigated 11 CSF cytokines in patients with CFS. They found that the levels of granulocyte-macrophage colony-stimulating factor reduced in the patients, while the levels of IL-10 increased. These results support the hypothesis that some symptoms of CFS occur due to immune system dysfunction within CNS [78]. However, subsequent studies yielded conflicting results. In a pilot study examining the role of 27 CSF cytokines in patients with CFS, the authors found that only IL-10 significantly decreased in the patients, and no significant differences were observed in other cytokines when compared with healthy controls. This observation was attributed to a weakened anti-inflammatory role of IL-10 in CNS [79]. Hornig et al. compared the levels of CFS cytokines among patients with CFS, patients with multiple sclerosis, and healthy controls. They found a markedly disturbed immune signature in patients with CFS, including disrupted IL-1 signaling and increased eotaxin and CXCL10 levels [80]. Additionally, not all CFS cases have a typical presentation; CSF cytokine levels exhibit different associations between typical and atypical CFS [81]. Results between different studies were heterogeneous due to the different experimental conditions and designs. Since the status of BBB, circulating cytokines, cytokines in CNS, as well as their interactions, are quite complex and different in different case, it is easy to understand the difficulty to use such cytokines in CSF as an independent index for CFS. Indeed, although the current body of evidence cannot support the use of cytokines in CSF as an independent biomarker for CFS. We believe that analysis of the cytokines in CSF, and combined consideration of the changes of circulating cytokines, must be helpful to understand the pathophysiological states of one CFS patient (such as inflammatory states of CNS, the state of BBB, etc.). Although the invasive nature of the procedure required to analyze the cytokine profile in CSF (lumbar puncture) may limit its clinical applications, comprehensive consideration the changes of CNS and circulating cytokines might be a selective strategy for diagnosis/assessment of CFS, which should be verified in the future.

## Cytokine modulation intervention for patients with CFS

Few clinical trials have demonstrated effective treatments for CFS. Exercise therapy is the most widely used treatment approach for CFS, but its efficacy remains controversial. Moreover, effective pharmacotherapies for CFS are currently lacking. Because there are similar clinical manifestations between sickness behavior and CFS, it has been suggested that CFS has neuro-immunological origins, where cytokine dysregulation may play a key role. Treatments to correct abnormal cytokine activities are, thus, considered to be beneficial for improving or curing CFS. Kerr et al. have suggested that randomized controlled trials (RCTs) should be designed to investigate the effects of IFN-β and a TNF-α inhibitor (e.g., etanercept), as IFN-β is effective for reducing fatigue in patients with multiple sclerosis. Additionally, TNF-α inhibitors have been proven to be effective in improving chronic inflammatory diseases such as arthritis and Crohn’s disease [82]. Arnett et al. have reported that anti-TNF drugs (e.g., infliximab and etanercept) may be effective in CFS patients due to their anti-inflammatory properties [83]; furthermore, they have reported that a combination of TNF-binding protein, IL-1 receptor antagonist, and anti-IL-6 monoclonal antibody might be effective for treatment of CFS [15]. Despite the prominent role of IL-1 in inducing fatigue, an RCT failed to find evidence for the efficacy of anakinra, an IL-1 receptor antagonist, in CFS patients [60]. On the basis of this finding of insufficient effective treatment against CFS, we suggest that more treatments to regulate abnormal cytokine expression should be developed and clinically tested.

## Concluding remarks

In the present review, we examined the roles of circulating cytokines and cytokines in CSF as potential diagnostic biomarkers for CFS. Furthermore, we discussed the potential value of such cytokines as therapeutic targets. It has been well documented that circulating cytokines such as TNF-α, IFN-γ, IL-6, and IL-1 are associated with CSF; however, many pathophysiological and methodological limitations prevent the use of such circulating cytokines as independent diagnostic markers. Moreover, there is no evidence to support the use of cytokines in CSF as independent indices. We believe that a comprehensive consideration of the changes in individual circulating and CNS cytokines may be a better approach for understanding the pathophysiology of CFS. In addition to other factors, such as clinical manifestation and duration of symptoms, changes in cytokine levels must be helpful in establishing an appropriate diagnosis; furthermore, cytokines can aid in subgroup classification.

With respect to the value of cytokines as therapeutic targets, the currently available evidence does not support the efficacy of interventions involving cytokine modulation. However, we believe that with an improved understanding of cytokine-related mechanisms, more appropriate therapeutic targets will be identified and closer associations will be confirmed between CFS and other neuroimmune disorders.

## Data Availability

The data and materials used are included in the review.

## Abbreviations

BBB: blood–brain barrier
CFS: chronic fatigue syndrome
CSF: cerebrospinal fluid
CNS: central nervous system
GM-CSF: granulocyte-macrophage colony-stimulating factor
HPA: hypothalamic–pituitary–adrenal
IL: interleukin
IL-1RA: IL-1 receptor antagonist
IFNs: interferons
ISF: insufficient symptoms or fatigue
LTα: lymphotoxin-alpha
MS: multiple sclerosis
NK: natural killer
PAI-1: plasminogen activator inhibitor
PBMCs: peripheral blood mononuclear cell
SB: sickness behavior
sFasL: soluble Fas ligand
TGF-β: transforming growth factor-beta
Th2: T helper 2
TNF-α: tumor necrosis factor-α
